# Is the surgical community prepared to face patients with SARS-CoV-2-induced cell death and organ injury in the post-pandemic era?

**DOI:** 10.1093/burnst/tkad049

**Published:** 2023-11-22

**Authors:** Sanketh Rampes, Sufia Ruhomaun, Qiang Shu, Daqing Ma

**Affiliations:** Division of Anaesthetics, Pain Medicine and Intensive Care, Department of Surgery and Cancer, Faculty of Medicine, Imperial College London, Chelsea and Westminster Hospital, London, UK; Division of Anaesthetics, Pain Medicine and Intensive Care, Department of Surgery and Cancer, Faculty of Medicine, Imperial College London, Chelsea and Westminster Hospital, London, UK; Department of Thoracic and Cardiovascular Surgery, The Children’s Hospital, Zhejiang University School of Medicine, National Clinical Research Center for Child Health, Hangzhou, China Hangzhou, China; Division of Anaesthetics, Pain Medicine and Intensive Care, Department of Surgery and Cancer, Faculty of Medicine, Imperial College London, Chelsea and Westminster Hospital, London, UK; Department of Thoracic and Cardiovascular Surgery, The Children’s Hospital, Zhejiang University School of Medicine, National Clinical Research Center for Child Health, Hangzhou, China Hangzhou, China

Coronavirus disease 2019 (COVID-19) has caused >760 million SARS-CoV-2 infections and 6.9 million deaths globally as reported by the World Health Organization (WHO). The virus is predominately a respiratory virus, but it has well-established effects on many organs, producing both short-term and long-term sequelae [1]. Studies early in the pandemic showed that perioperative COVID-19 was associated with significant increases in postoperative complications and mortality. This led to governing medical organizations in the UK recommending that elective surgery should be delayed 7 weeks after COVID-19 infection, and possibly longer for those with ongoing symptoms at 7 weeks [1,2]. COVID-19 presents novel challenges to patients and the surgical community worldwide, highlighting the need for renewed focus on perioperative care.

Perioperative medicine is the practice of patient centred, multidisciplinary and integrated medical care throughout the entire surgical journey. An essential aspect of perioperative medicine is the systematic evaluation of the functioning of vital organs, as well as their optimization prior to surgery; evidence shows preoperative evaluation reduces postoperative complications, delirium, 30-day mortality and duration of hospital stay [3]. A comprehensive assessment of all body systems is all the more pertinent in the context of COVID-19, as emerging research details the effects of the virus on multiple organs and the long-term sequalae that ensue [1]. The post COVID-19 condition, colloquially referred to as long COVID, has been outlined by a consensus led by the WHO as a minimum 2 months of new onset symptoms after initial recovery from acute COVID-19 or ongoing symptoms from the initial illness [1]. Many symptoms and conditions have been reported and, in particular, ongoing respiratory function impairment is readily noted [4]. Biochemical and radiological changes attributed to COVID-19 have been documented, e.g. a UK-based research group used magnetic resonance imaging to demonstrate mild organ impairment 4 months after acute COVID-19 infection in 70% of their cohort (n = 201), 29% of whom had more than one organ affected [5]. Proposed mechanisms underpinning the multisystemic and long-term effects of COVID-19 include SARS-CoV-2 tissue reservoirs, immune dysregulation, abnormalities in clotting and endothelium, microbiota dysbiosis and dysfunctional neurological signalling [4]. Much of this research is in its infancy, highlighting the importance of thorough preoperative evaluation of all body systems as future work continues to substantiate the widespread effects of COVID-19 on the body.

Perioperative infection with COVID-19 was associated with clinically significant increases in mortality, with some studies reporting a >10-fold increase [2]. The COVIDSurg Collaborative designed a large international prospective cohort study to identify the optimal amount of time by which surgery should be delayed in patients with COVID-19 infection; therefore 30-day mortality outcomes were compared in surgical patients with and without preoperative SARS-CoV-2 infection. The odds ratio (OR) of mortality was higher in the preoperative COVID-19 groups where the surgical intervention occurred 0–2 weeks [4.1; 95% confidence interval (CI) 3.3–4.8], 3–4 weeks (3.9; 95%CI 2.6–5.1) and 5–6 weeks (3.6; 95%CI 2.0–5.2) after infection [6]. Patients having surgery >7 weeks after COVID-19 infection had no increased post-operative mortality compared to the group without preoperative infection. However, within this cohort of patients having surgery >7 weeks after COVID-19 infection, those with ongoing symptoms had a higher mortality rate compared to those whose symptoms had resolved or who were asymptomatic [6]. Other studies have largely supported these findings; a large retrospective case-control study looked at high-risk operations in the USA between January 2020 and March 2021 to determine the safest time to operate after COVID-19 infection as measured by 90-day all-cause postoperative mortality [7]. The study found that patients who underwent a major operation within 8 weeks of a positive diagnosis of COVID-19 had a higher 90-day postoperative mortality than matched-controls without a diagnosis of COVID-19; those that had an operation performed within 7 to 8 weeks after the positive tests had a mortality of 12.3% compared to 4.9% in the matched cohort [7].

Since the publication of the above recommendations, more recent studies have specifically investigated the impact of the COVID-19 vaccine and timing of surgery following COVID-19, as well as the impact of the omicron variant on post-operative mortality. A large retrospective cohort study focused on whether COVID-19 vaccination status modified major postoperative complications within 30 days of surgery in patients who underwent elective operations in northern California from January 2020 to February 2022, stratifying the patients by time of operation relative to COVID-19 diagnosis [8]. The study found that there were increased postoperative complications in unvaccinated or partially vaccinated patients who underwent surgery within 0 to 4 weeks after a positive COVID-19 diagnosis. However, there was no increase in postoperative complications in fully vaccinated patients who had been diagnosed with COVID-19 infection prior to their operation or in unvaccinated or partially vaccinated patients who had operations 4 to 8 weeks post COVID-19 infection [8]. Another large population based-cohort study, published after the COVIDSurg Collaborative findings, aimed to determine whether there is a relationship between previous COVID-19 infection and adverse outcomes (measured as the composite risk of death, major cardiovascular events and rehospitalization within 30 days) following elective major noncardiac surgery. The work indicated that previous COVID-19 infection within 6 months of surgery was not associated with adverse outcomes; furthermore this conclusion was also reached in patients who had surgery within 4 weeks of infection (Adjusted relative risk-aRR, 1.15; 95%CI, 0.64–2.09) and <7 weeks after infection (aRR, 0.95; 95%CI, 0.56–1.61) [9]. Again, these discrepancies are attributed to the changing COVID-19 landscape and vaccinated population. Taking these factors into account, a large prospective study in France used a highly contemporary omicron-predominant and largely vaccinated cohort of patients undergoing scheduled surgery between March and May 2022 [10]. The data echo that of the more recent studies and no differences in postsurgical respiratory morbidity were seen between patients with preoperative COVID-19 infection within 8 weeks of operation and those without preoperative COVID-19 infection (OR 1.08, 95% CI, 0.48–2.13) [10].

As we navigate a post-pandemic population, the patient-centred, multidisciplinary and integrated approach of perioperative medicine will be integral when considering and optimizing individuals for surgical intervention. There needs to be renewed focus on preoperative assessments and perioperative care that must include COVID-19 infection history, considering possible organ impairment and ongoing symptoms in order to provide a comprehensive preoperative evaluation. In terms of optimizing individuals for surgery, the aforementioned multidisciplinary consensus suggests that vaccination several weeks before surgery will reduce postoperative mortality and complications of COVID-19 infection which is also supported by recent studies [2,8,10]. As of current, more robust and high-powered research building upon these findings is needed before recommendations are changed, and elective surgery should continue to be delayed by 7 weeks after COVID-19 infection unless the risks outweigh the benefits. However, as recommended by the consensus, we propose that rather than timing alone, there should be a strong focus on individual factors including baseline risk, rigorous perioperative assessment and preoperative optimization, addressing modifiable risk factors such as nutrition, pre-operative exercise and smoking cessation, alongside shared-decision making with patients when deciding on the timing of elective surgery [2].
